# Contemporary management of stage i testicular seminoma: a survey of Canadian radiation oncologists

**DOI:** 10.3747/co.v15i4.213

**Published:** 2008-08

**Authors:** R. Samant, I. Alomary, P. Genest, L. Eapen

**Affiliations:** * Radiation Oncology Program, The Ottawa Hospital Cancer Centre, and Faculty of Medicine, University of Ottawa, Ottawa, ON.

**Keywords:** Stage i seminoma, treatment, survey, radiation oncologists, surveillance, radiotherapy, chemotherapy

## Abstract

Recently published studies clearly indicate that there are now several acceptable options for managing stage i testicular seminoma patients after orchiectomy. We therefore decided to survey Canadian radiation oncologists to determine how they currently manage such patients and to compare the results with previous surveys.

Our results demonstrate that adjuvant single-agent chemotherapy is being considered as an option by an increasing proportion of radiation oncologists (although it is not considered the preferred option), the routine use of radiotherapy is declining, and surveillance is becoming increasingly popular and is recommended most often.

## 1. INTRODUCTION

Testicular cancers are the most common—and the most curable—malignancies among young men in North America 1,2. Seminomas account for approximately half of these cancers, and most patients (80%) present with stage i disease 3,4. Treatment is highly successful, with 5-year overall and disease-specific survivals approaching 100% for stage i seminoma 2,5.

For decades, the standard treatment for this cancer has been radical inguinal orchiectomy, followed by adjuvant radiotherapy to the para-aortic and ipsilateral pelvic regions 2,5,6. This approach led to a recurrence rate of less than 5%, with salvage chemotherapy being highly effective in the few patients that did relapse 2,3,5,6. However, long-term follow-up data (beyond 10–15 years) now indicate that treatment-related morbidity and mortality (especially from a second malignancy) are significant concerns 3,7,8. Various approaches have therefore been investigated to minimize the toxicity associated with routine use of radiotherapy. One approach has been to minimize toxicity by reducing radiotherapy field sizes and doses 9,10; another approach has been to avoid radiation altogether 3. This change has, in turn, resulted in the evolution of surveillance after orchiectomy as a viable option because of the general availability of computed tomography (ct) imaging for follow-up purposes 11. In addition, the use of single-agent chemotherapy (most commonly 1–2 cycles of carboplatin) has also been recognized as a potential option in place of radiotherapy 12–14.

As a result, management of stage i seminoma is currently focused not only on maintaining high rates of cure, but also minimizing both short- and long-term toxicity 3,11,15. Previously, a survey of radiation oncologists conducted by Choo *et al.* in 2001 regarding management of stage i seminoma revealed considerable variation in practice, especially with regard to treatment 16. Most radiation oncologists at that time routinely discussed surveillance as an option, but thought that only 20% of patients would choose that option. At that time, the authors found that adjuvant radiotherapy was usually the preferred choice. However, data have continued to accumulate regarding the viability of options that do not include the routine use of adjuvant radiotherapy.

Review of seminoma management at our own institution 17 showed a steady decline in the use of adjuvant radiotherapy since the 1990s and the increasing use of surveillance for stage i seminoma patients—a trend that has also been reported elsewhere 18,19. Although recent studies suggest that the use of single-agent chemotherapy with carboplatin is very encouraging and the use of adjuvant chemotherapy is becoming increasingly popular in Europe 20, this approach is still not routinely considered at our institution. Our experience has been that chemotherapy tends to be reserved for the salvage of radiotherapy failures or in patients wanting adjuvant treatment who are not deemed suitable for radiation. Based on the increasing options for management of stage i seminoma, we decided to survey Canadian radiation oncologists to see if their management approaches had changed since earlier in the decade.

## 2. METHODS

We developed an electronic survey to assess management of stage i testicular seminoma patients. The survey was specifically designed for radiation oncologists, and the categories evaluated included staging investigations, treatment options, radiotherapy treatment planning details, surveillance protocols, and respondent demographic information. Intended for self-completion, the survey takes approximately 15 minutes to finish. After obtaining approval from the research ethics board of the Ottawa Hospital to proceed with this survey study, we sent the survey by e-mail in 2005 to Canadian radiation oncologists identified as treating genitourinary malignancies. The list of radiation oncologists was formulated from information obtained from the directory of the Canadian Association of Radiation Oncologists at that organization’s Web site and from correspondence with radiation oncology department heads at cancer centres across Canada. Initial non-responders were sent reminder notices, also by e-mail. Remuneration was not offered for completing the survey.

The completed surveys were collated and analyzed for this study. The chi-square statistic was used to assess associations between the respondent’s practice history and that person’s responses to survey questions. To compare differences in choice of treatment attributable to age and years of practice, a test of mean differences was applied using analysis of variance. The SPSS software package (SPSS, Chicago, IL, U.S.A.) was used to perform the analyses.

## 3. RESULTS

Electronic surveys were sent to 119 Canadian radiation oncologists, and 93 responses were received (78% response rate). Of the respondents, 14 indicated that they did not treat seminoma patients, and 1 declined to complete the survey. The survey completion rate among eligible responders was therefore 74% (78/105). Among respondents completing the survey, 89% were men and 11% were women. Median age was 43 years. Mean length of practice was 13 years, and 80% of the respondents worked in academic centres.

**FIGURE 1:**
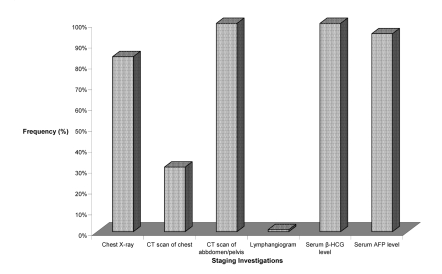
The frequency of specific staging investigations used for stage i seminoma patients, from a sample of Canadian radiation oncologists. ct = computed tomography; afp = alpha-fetoprotein; β-hcg = beta–human chorionic gonadotropin.

Figure 1 shows that the staging investigations most commonly used are ct scans of the abdomen and pelvis (100%) and chest radiographs (84%). Serum tumour markers are also commonly assessed, with alpha-fetoprotein (afp) being measured 95% of the time, and beta–human chorionic gonadotropin (bhcg) levels 100% of the time.

**FIGURE 2:**
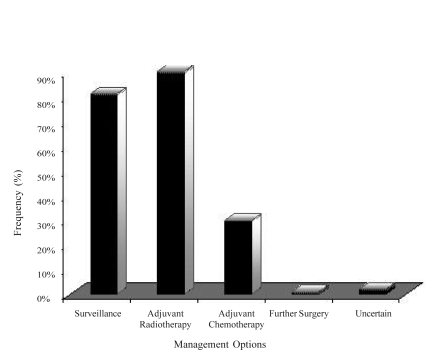
Standard management options used for stage i seminoma patients, from a sample of Canadian radiation oncologists.

**FIGURE 3:**
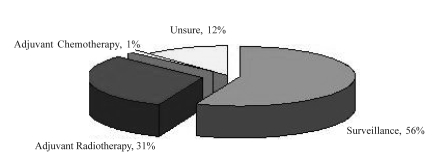
The “most appropriate option” for most stage i seminoma patients, from a sample of Canadian radiation oncologists.

Adjuvant radiation and surveillance were considered the most common standard treatment options by 90% and 81% of respondents respectively. However, 30% of respondents also listed adjuvant chemotherapy as a standard treatment option (Figure 2). However, when asked to indicate the management that they felt was most appropriate for most patients, surveillance was chosen by 56%; radiotherapy, by 31%; and chemotherapy, by 1%. The remaining 12% were unsure of the most appropriate management (Figure 3). The most common concerns related to the use of adjuvant radiotherapy were second cancers (84%), infertility (77%), late gastrointestinal toxicity (67%), acute nausea and vomiting (61%), and late renal toxicity (60%).

Most respondents (91%) said that they routinely discuss surveillance with patients, but that tumour-related risk factors (size, local extension, and lymphovascular invasion), together with patient age and compliance, influence their recommendations. Nearly all respondents (<95%) started offering surveillance during the last 10 years. Most respondents felt that at least 50% of patients are now choosing surveillance. The most commonly listed reasons, in order of importance, for not offering surveillance were patient fears and anxieties, patient reluctance, increased costs, and the belief that survival was actually better with the use of adjuvant radiotherapy.

For patients on surveillance protocols, the investigations commonly used are ct scans of the abdomen and pelvis (93%), chest radiographs (81%), bhcg levels (92%), and afp levels (84%). Surveillance investigations are usually carried out every 3–4 months by 84% of respondents, every 6 months by 15%, and every 12 months by 1%.

When treating with radiotherapy, 50% use para-aortic fields only, and 50% use para-aortic and ipsilateral pelvic fields. Planning ct is used for simulation by all respondents (100%); intravenous pyelograms and lymph angiograms are used by only 1%. A dose of 2500 cGy in 15–20 fractions over 3–4 weeks was recommended by more than 90% of respondents, and all respondents use linear accelerators (≥ 6 MV photons) for treatment delivery. Scrotal shielding is routinely used by 49% of respondents to reduce dose to the contralateral testicle, and thermoluminescent dosimeters are used by 47% for verifying dose to the contralateral testicle. Almost all respondents (96%) discussed fertility issues and sperm banking with patients before the start of adjuvant radiotherapy.

Prophylactic antiemetics are ordered by 58% of the respondents, with ondansetron, prochlorperazine, dimenhydrinate, and metoclopramide being prescribed by 48%, 25%, 18%, and 8% respectively. Following treatment, 67% recommend that patients take contraceptive measures for at least 3 months (14%), 6 months (38%), 12 months (40%), or 24 months (8%).

We observed a trend, although not statistically significant, for older radiation oncologists (> 45 years vs. ≤ 45 years) to choose radiation for their patients (*p* = 0.07). However, years in practice, type of practice (academic vs. community), and provincial location did not appear to influence management choices.

## 4. DISCUSSION

Cancer treatment approaches evolve with time and accumulation of research findings—first to improve and maximize cure rates, and then to minimize toxicity. Stage i seminoma management certainly confirms this paradigm. The use, since the 1960s, of adjuvant radiotherapy after orchiectomy reduced relapse rates to less than 5% and established adjuvant radiation as the standard practice until the 1990s 2,6. In fact, overall 5-year survival rates were in the 98%–99% range, because chemotherapy was highly successful in salvaging the few patients that did relapse post  radiotherapy 20. However, with the accumulation of long-term follow-up data over 25 years or more, it became obvious that second malignancies are a significant problem following radiation 3,7. The prevailing focus therefore moved to reduction of toxicity, because salvage therapies were so effective 3,6.

This change in focus led to numerous studies evaluating alternatives to standard radiotherapy, and reductions in radiotherapy treatment volumes and lower radiation doses were both shown to be possible without significantly reducing efficacy 9,10. In prospective and retrospective studies, surveillance approaches have also been shown to be an excellent alternative that do not compromise cure rates, although they require more frequent imaging investigations 11,17–19. More recently, the use of adjuvant single-agent chemotherapy (most commonly with single-agent carboplatin) has been shown to yield results, in terms of relapse rates and overall survival, similar to those seen with the use of adjuvant radiation 14. Therefore, currently, there is evidence that all of the above approaches are effective and reasonable options that have their own unique advantages and limitations. However, long-term follow-up data for the newer approaches are limited, and questions still exist regarding long-term efficacy and toxicity.

The effect of recent studies and their influence on actual current practice patterns across North America and Europe has not been fully evaluated. However, it is well known that medical practice patterns often change gradually after research findings are published, as physicians reflect on the available evidence and perhaps discuss them with their colleagues 21–23. Therefore, we believe that it is important to determine whether the accumulating evidence regarding management of stage i testicular seminoma has had an effect at the level of clinical practice in Canada, and that is what we attempted to do in the present study.

With a very favourable survey completion rate of 74% 24, we believe that our results generally reflect current opinion among Canadian radiation oncologists regarding stage i seminoma management. Our findings show that staging is fairly consistent and reflects the emergence of ct scans of the abdomen and pelvis as standard, together with chest radiographs 11 and serum tumour markers (afp, bhcg). Radiation treatment planning with ct scans and delivery approaches using linear accelerators, as indicated by the respondents, are also consistent with the published literature, as are the dose and fractionation regimens commonly used 5. However, opinion continues to vary regarding the necessity of ipsilateral pelvic lymph node irradiation.

The surveillance protocols among respondents are very similar to published recommendations 15. Although variations in practice still occur, a preponderance of respondents indicate that they routinely discuss both surveillance and adjuvant radiotherapy with their patients, and most believe that surveillance is the preferred option, with at least 50% of patients making the latter choice.

Adjuvant chemotherapy is also starting to be recognized as a treatment option by almost one third of radiation oncologists even though our survey was conducted before the results of the Medical Research Council E19 study, published by Oliver *et al.* 14, indicated that at a median follow-up of 4 years, adjuvant single-agent carboplatin chemotherapy was essentially equivalent in terms of relapse rates and overall survival to adjuvant radiotherapy. Our finding in this regard represents a major shift from the survey results reported in 2002 by Choo *et al.* 16, when most radiation oncologists thought that adjuvant radiotherapy should be standard, although surveillance was considered an option. Also, chemotherapy was not even considered an option by any respondents at that time, likely reflecting the limited published data to that point.

The biggest issue remains the attempt to achieve a balance between minimizing relapse rates and patient fears and anxiety related to recurrences, and avoiding unnecessary treatment for the preponderance of patients that will not relapse and the potential for toxicity that can occur decades later. This balance will likely remain controversial for some time to come, and it is uncertain whether consensus can be achieved in the near future. Individual patient factors, including personal choice, will also be essential in determining the management option that is chosen. However, it is reassuring to confirm that many radiation oncologists have been re-evaluating their approach to management of stage i testicular seminomas over the last 5 years, in parallel with growing evidence of newer management approaches. This re-evaluation may in part be attributable to the growing sub-specialization among many practicing radiation oncologists in terms of site-specific treatment and the resulting familiarity with recently published studies, and also to the Canadian sources of much of the published literature regarding surveillance for management of stage i seminoma 11,17–19. We expect that management approaches will continue to evolve as ongoing studies mature, especially those evaluating the use of single-agent carboplatin chemotherapy.

## 5. CONCLUSIONS

Canadian radiation oncologists are routinely discussing surveillance in addition to adjuvant radiotherapy as treatment options for patients with stage i testicular seminoma, but surveillance is usually considered the preferred option. Chemotherapy also appears to be emerging as a viable option among a growing number of radiation oncologists. As a result, the use of radiation is declining.
